# Gender Wage Gap and Male Perpetrated Child Sexual Abuse

**DOI:** 10.21203/rs.3.rs-2857277/v1

**Published:** 2023-04-28

**Authors:** Zainab Hans, Michael Belzer

**Affiliations:** University of Michigan–Ann Arbor; Wayne State University

**Keywords:** Child Sexual Abuse, Wages, Employment, Intra-household bargaining, J13, J12, I10

## Abstract

Given the fact that child abuse and intimate partner violence often co-occur, intra-household bargaining models provide a useful framework to investigate the relationship between macro-economic factors and child sexual abuse (CSA). Non-cooperative bargaining models predict that labor market opportunities that benefit women improve their bargaining power and lead to lower levels of intimate partner violence against them. We posit that this protective effect extends to children as well, and exploit exogenous variation in macro-economic factors to examine the impact of gender specific wages and employment on police reported CSA in South Carolina, Tennessee, and Virginia from 2006 to 2019. The empirical analysis provides evidence that narrowing the gender wage gap leads to a decline in police reported CSA incidents perpetrated by mothers’ intimate partners, whereas improvements in relative employment opportunities do not yield any such effects. Consistent with previous literature, our results show that wages, not employment, determine bargaining power. The findings also underscore important spillover benefits of policy solutions directed towards narrowing the gender wage gap.

## 1. Introduction

According to the United States Department of Health and Human Services, nearly 4 million reports of child maltreatment are filed across the country each year. Of these, approximately 674,000 cases of child maltreatment and neglect are substantiated annually (U.S. Department of Health & Human Services et al., 2019). The fourth National Incidence Study of Child Abuse and Neglect (NIS-4) estimated that 1 in 58 children was a victim of maltreatment in 2005–2006. Estimates of self-reported maltreatment based on surveys, on the other hand, range from 23–38% (Currie & Tekin, 2012; Finkelhor et al., 2015). While all forms of maltreatment can lead to adverse outcomes, abuse specifically has significant repercussions for young victims (Nemeroff, 2016, Fry et al. 2018). Among children who experience it, 58% are subjected to physical abuse and 24% are subjected to sexual abuse (Finkelhor et al., 2015). A recent meta-analysis showed that prominent risk factors for child sexual abuse (CSA) include intimate partner violence (IPV), exposure to physical child abuse, and presence of a non-biological parental figure (Assink et al., 2019). Violence in the home (Adhia et al., 2019; U.S. Department of Health & Human Services et al., 2019) and poverty (Berger, 2005; Bullinger et al., 2022; Paxson & Waldfogel, 2002; Slack et al., 2004) also put children at risk for physical abuse. Previous investigations into the relationship between child welfare and economic conditions show that indicators of financial stress experienced by parents such as mortgage foreclosure, delinquency rates (Wood et al., 2012), and residential insecurity (Bullinger & Fong, 2021) are associated with child maltreatment. An inverse relationship between child abuse and parental employment (Frioux et al., 2014; Raissian, 2015; Schneider et al., 2021) and income (Brown & De Cao, 2020; Cancian et al., 2013; Raissian & Bullinger, 2017) has also been noted.

This body of literature highlights several mechanisms through which macro-economic factors can affect children’s welfare. Higher wages and stable employment can lower child abuse by relieving financial burden on families, and attenuating stress related triggers that can prompt violence. While the stress relieving mechanism of improved labor market opportunities provide an explanation for reduction in physical child abuse, it is an unlikely explanation for CSA that occurs within households. Sexual victimization of children is predatory and coercive (Craven et al., 2006; Katz & Barnetz, 2016), and its pathways differ from that of physical violence (Winters & Jeglic, 2017). Therefore, the link between larger economic macro-economic factors and CSA remains unclear.

Fortunately, there has been a noted decline in CSA since the 1990s (Finkelhor, 2020; Finkelhor et al., 2018). This is often attributed to prevention efforts that have centered around initiatives focused on raising awareness, encouraging disclosure, and offender management. However, a dearth of empirical evidence makes it difficult to ascertain the effectiveness of these interventions in reducing CSA (Finkelhor, 2009). Given that IPV and child abuse frequently co-occur (Adhia et al., 2019; Bidarra et al. 2016; Fry & Elliott, 2017), we employ an intra-household bargaining framework to show that improvements in women’s economic prospects are protective against CSA perpetrated by mothers’ male partners. While previous studies have extended the intra-household bargaining framework and provided theoretical justifications for a negative relationship between child victimization and mothers’ bargaining power (Berger, 2005), causal inferences have been hampered by data constraints. Our study fills this gap by presenting the first empirical evidence of a causal relationship between macro-economic factors and police reported CSA. We construct a longitudinal panel of CSA perpetrated by biological fathers and mothers’ male intimate partners using data from the National Incident Based Reporting System (NIBRS). Employing a well-established strategy to exploit exogenous variation in gender specific labor market opportunities, we demonstrate a causal relationship between mothers’ bargaining power and male perpetrated CSA reported to law enforcement.

Since literature on intra-household bargaining highlights that wages, not employment, determine bargaining power at threat point (Pollak, 2005), we consider both in our analysis. The results show that narrowing of the gender wage gap leads to a reduction in police reported CSA but improvements in relative employment opportunities yield no such effects. Our study makes several unique and valuable contributions to the existing literature. First, it shows that improvements in women’s bargaining power have a protective effect on children and as such identifies a powerful primary prevention tool for policy makers. Second, it highlights that in studying macro-economic factors associated with child abuse it is important to consider the mechanisms through which these factors impact family interactions within households. While wages and employment may appear analogous as measures of women’s economic empowerment, their effects are not equivalent, and these differences should inform policy decisions.

## 2. Intra-household Bargaining and Child Wellbeing

Existing evidence indicates that child outcomes improve when mothers have greater bargaining power (Ahmed, 2006; Echeverría et al., 2019). Greater control over household finances by women is related to lower levels of mortality and morbidity in children (Thomas, 1990), as well as increased in expenditures on necessities such as clothing (Lundberg et al., 1997). Female autonomy and empowerment are also linked to improvements in children’s anthropometric status (Heckert et al., 2019; Malapit et al., 2015). These findings suggest that mothers are more invested in children’s health and wellbeing and leverage their bargaining power to facilitate better outcomes for their offspring.

There is also compelling evidence to show that IPV decreases in response to improvements in women’s economic status (Aizer, 2010; Anderberg et al., 2016; Bhattacharyya et al., 2011), indicating that women can use the increased bargaining power to negotiate lower levels of violence against themselves. A natural extension of these models, where a woman’s utility function incorporates her child’s wellbeing, implies that the protective effect will extend to the child as well. There are two plausible channels through which this protection may be operationalized. Economic empowerment can equip mothers with resources necessary to leave abusive relationships which can reduce children’s exposure to abusive adults. Favorable economic prospects can also endow mothers with greater bargaining power which can be leveraged to ensure children’s safety.

## 3. Material and Methods

### 3.1. Measures of Child Sexual Abuse

Measures of CSA used in this study are constructed using the National Incident Based Reporting System (NIBRS) data from 2006 to 2019. NIBRS extracts data from police incident reports and contain rich demographic information on victims and offenders, as well as information on the relationship between the two. Law enforcement agencies (LEAs) voluntarily opt into the NIBRS system. Consequently, data is not available for all jurisdictions. There is also temporal variation in NIBRS adoption across agencies, therefore, to create a stable panel of police reports, three states, South Carolina; Tennessee; and Virginia, are included in the analysis.

Comparing child abuse cases from NIBRS to those reported by the child welfare system in South Carolina, Finkelhor, and Ormrod found that the number of CSA cases reported by both systems were the same (Finkelhor & Ormrod, 2001). Tennessee law requires immediate notification of sexual abuse cases to the district attorney and the judge. Virginia has similarly enacted statutes that require law enforcement to be notified of all incidents of actual and suspected CSA reported to child protective services (Virginia Department of Health and Human Services). As such, NIBRS data likely captures a vast majority, if not all, of CSA reported to authorities in these states...

Utilizing information on the victim’s age and relationship to the perpetrator, county-year measures of CSA perpetrated by biological fathers, stepfathers, and mothers’ intimate partners were created by aggregating the following offenses: forcible rape; forcible sodomy; sexual assault with an object; and forcible fondling for victims under 18. To avoid differential reporting across time and space, we include agencies that reported 12 months of data for each year in our study but include agencies that submitted their data to NIBRS through another agency but later became direct contributors. Relationship information was missing in about 15% of all CSA incidents reported by NIBRS. This was imputed using information on victim and incident characteristics. Of the total CSA incidents perpetrated by biological fathers and mother’s intimate partners, 3.2% were reported by state police and other federal agencies over the analysis period. These were excluded since the origination county of these reports cannot be determined.

Since there may be important limitations inherent in the bargaining process as biological fathers’ parental rights do not terminate upon the dissolution of relationship with the mother, it is important to distinguish between relationship categories to avoid obscuring important differences. We estimate the models for each category separately. For ease, we group stepfathers and mothers’ dating partners together into a single category. Additional measures of sexual assault perpetrated by family members (such as siblings, grandparents, other related individuals) and strangers are employed for comparison. While as a rule NIBRS does not contain unfounded cases, sexual assault is an exception and a small percentage of cases may be determined to be unfounded (Kaplan, 2021). However previous studies examining reported cases of child abuse show that risk to children is similar in both instances (Kohl et al., 2009) and that a large proportion of subsequent reports are substantiated after an initial denial (Jedwab et al., 2017).

### 3.2. Control Variables

The choice of control variables is informed by literature. Previous research shows that factors like prosecution and probability of conviction can deter perpetration (Loughran et al., 2016). However publicly provided services, like the prosecutor’s office, suffer from greater inefficiencies that are associated with the economic status of the county within which they operate (Gorman & Ruggiero, 2009). If improvements in economic conditions lead to greater resources and thereby higher conviction rates which discourages perpetration, than omitting controls to capture this effect can bias the estimated effect of our variable of interest on child abuse. We include the Yost Index to control for socio-economic differences between counties. Yost index is a composite measure of median household income; median rent; median home value; percentage of population living below 150% of the poverty line; percentage of population that is unemployed; percentage of population employed in blue collar jobs; and an education index (Yost et al., 2001). Disadvantaged communities and communities with high crime rates often receive more scrutiny by law enforcement and are policed aggressively (Edwards, 2019). Families engaged in the welfare system are also more likely to be involved with child protective services (Berger et al., 2009). It is important to account for disproportionate exposure and scrutiny that may arise because of involvement in the welfare system. We include a log of total households receiving Supplemental Nutrition Assistance Program (SNAP) in each county to control for such exposure. If reporting and/or perpetration is affected by the size of the police force, then one also needs to control for it (Anderberg et al., 2016; Lindo et al., 2018). We include the log of total number of police officers in each county using FBI’s Law Enforcement Officers Killed and Assaulted (LEOKA) data.

High rates of violent crime can also trigger stress in the residents of a community which can contribute to intrahousehold violence (Beyer et al., 2015; Wallace et al., 2021). This environmental effect is approximated by including a variable that captures the occurrence of violent crimes (aggravated assault, rape, robbery, and murder) in each county. Since IPV and child abuse tends to co-occur, we also include the log of county-year aggregates of IPV assaults calculated from NIBRS data.

Given the significant variation in county sizes and their respective populations, we use county level population data, for children under 18, from the National Cancer Institute’s Surveillance, Epidemiology, and End Results (SEER) program to include proportion of children in each county that is white, black, or other minority race as controls and use the total child population as an offset. Finally, we use county to county migration data from the internal revenue services (IRS) to proxy the in-migration of individuals to each county to control for the possibility of counties with better economic conditions attracting individuals who have lower propensity to commit CSA (e.g. better educated Individuals). Descriptive statistics are provided in Table 1.

### 3.3 Empirical Strategy and Model

We construct two distinct measures of gender-specific labor market opportunities by exploiting statewide shifts in labor demand to capture exogenous changes (Aizer, 2010; Autor & Duggan, 2003; Bound & Holzer, 2000; Hoynes, 2000). County specific data on employment and wages for NAICS classified industries come from Quarterly Census of Employment and Wages (QCEW), Bureau of Economic Analysis, and the Census Bureau’s Equal Employment Opportunity data. For each county, we calculate industry specific annual wages by indexing statewide employment in an industry with the proportion of men and women employed within that industry in each county. This yields average wages earned by workers of each gender in that county per industry which are aggregated to generate county totals and calculate female to male wage ratio.

1 $$w_{gcy}=p_{gnc}.i_{ncy}$$

Where $p_{gnc}$ is the base period proportion of each gender employed in industry *n* in county *c*, and $i_{ncy}$ is the industry wage for each county *c* in year *y*. The base period shares of employment by each gender are held constant over the length of the analysis period to ensure that changes in wages are driven by the demand side. Therefore, gender specific wages vary depending on the relative concentration of industries within each county and whether these industries employ a larger proportion of male or female workers (Aizer, 2010).

Since time allocation to labor market can change at threat point if non-working individuals choose to obtain employment or employed individuals increase the number of hours worked, employment should not have a determinate impact on bargaining power (Pollak, 2005). We use predicted employment rates to confirm that changes in sexual victimization of children arise from changes in bargaining power. We construct gender specific employment rates following the same approach used in creating wages. The base period shares of statewide NAICS industry employment for each gender in each county are multiplied by total statewide industry employment and aggregated across all industries for each year.

2 $$e_{gcy}=q_{gnc}.l_{ny}$$

where $q_{gnc}$ is the base period share of statewide employment of each gender in industry *n* in county *c* and $l_{ny}$ is the total statewide employment in industry *n* in year *y*. Like wages, the variation in employment comes from changes in state level industry specific employment and differs across counties based upon the growth rates of each industry over the analysis period. The resulting employment counts are divided by annual gender-specific population counts obtained from Surveillance, Epidemiology and End Results Program data (SEER) for each county.

Our empirical strategy was motivated by the nature and features of the data. Our dependent variable is over-dispersed with a non-trivial number of zeros even after aggregation and exclusion of counties with very small populations from the analysis to increase statistical power. Additionally, dependencies can arise due to temporal hierarchies as well as larger legal and socio-economic constructs (Johnson, 2006). These methodological and theoretical concerns necessitate the use of a multilevel model to properly accommodate the hierarchical nature of the data. We employ a negative binomial model with a group mean centering approach to capture both the within-subject and between-subject variation among counties. In this specification, the effects of time-invariant unobservable effects are controlled by inclusion of independent time-varying covariates that are operationalized as county-specific means. Time varying effects are captured by deviations from the mean of the same covariates (Allison, 2005, 2009; Bell & Jones, 2015). We also include state and year fixed effects to control for any unobservable time-varying confounders as well as state-year linear time trends yielding the following functional form

$$log\left(\pi_{jct}\right)=\beta_{0}+\beta_{1}\left(x_{jct}-{\bar{x}}_{jc}\right)+\beta_{2}{\bar{x}}_{jc}+\beta_{3}\tau_{jt}+\beta_{4}\phi_{jt}+\left(\mu_{jc}+e_{jct}\right)+\log\left(p_{ct}\right)$$

where $x_{jct}$ is an explanatory variable belonging to county *c*, nested in state *j* measured at time *t*, ${\bar{x}}_{jc}$ is the vector of subject-specific means of the explanatory variables, $x_{jct}-{\bar{x}}_{jc}$ is the deviation from the mean, and $\mu_{jc}$ and $e_{jct}$ are the error terms associated with subjects and time periods respectively. This allows us to model the average between and within effects distinctly in one coherent framework while controlling for unobserved time-invariant characteristics for each county and ensuring that there is no correlation between ${\bar{x}}_{jc}$ and $x_{jct}$ by apportioning out $\mu_{jc}$. $p_{ct}$ is the offset that accounts for the variation in child population across the different counties; $\tau_{jt}$ is the vector of state-year fixed effects, $\phi_{jt}$ is the linear time trend.

## 4. Results

### 4.1. Impact of Wages on Child Sexual Abuse

Table 2 presents the coefficients from models estimating the impact of wage ratio on CSA by different relationship categories. Column 1 shows the results of sexual abuse perpetrated by biological fathers and column 2 shows sexual abuse perpetrated by mother’s intimate partners while columns 3 and 4 show abuse by other family members and by strangers respectively. Since the predominant household structure in the US comprises of a nuclear family and intra-household bargaining does not extend to strangers, models 3 and 4 serve as falsification checks for the primary models in column 1 and 2. Our main objective is to establish a causal relationship and obtain unbiased estimates of the effects of wage gap over time, therefore we primarily focus on the within wage ratio and restrict the interpretation of ${\bar{x}}_{jct}$ as differences between counties.

Looking at the within effects, wage ratio is negative and significant at the one percent level for mother’s partner, showing that as gender wage gap closes there is a statistically significant decline in police reports of CSA. The within effect of wage ratio is insignificant in columns 3 and 4, indicating that the protective effect of female wages is restricted to abuse that occurs within a household instead of some general mechanism that provides broader immunity to children and insulates them from sexual violence regardless of the source of perpetration. Wage ratio also does not have a significant effect on CSA by biological fathers. Exponentiating the coefficients in column 2 and interpreting them as incidence rate ratios show that a one unit increase in the wage ratio reduces police reports of child sexual abuse by a factor of 0.11 (IRR 0.11, 95% C.I. 0.027–0.456). Admittedly a one unit increase in wage ratio is not intuitive. Estimating the effect of one standard deviation in wage ratio yields 0.64 (or 23%) fewer incidents of CSA when the wage ratio rises from 0.75 to 0.87. The between effects in the first three models show that on average counties with higher gender wage ratios have lower levels of CSA but not significantly so.

Additional within effects show that, increases in IPV are positively and significantly associated with CSA by biological fathers, mothers’ partners, and family members. The between coefficient of IPV directed towards females is also positive and significant in models 1, 2, and 3. This is consistent with prior research that shows that IPV and child abuse tend to co-occur. The between coefficients of secular violence is positive and significant in all models except for mother’s partners, while the within coefficient is significant in all models. This indicates that children living in areas with higher rates of overall violence are more vulnerable to sexual exploitation and increases in violence in a community are associated with an increase in CSA.

The results of the primary specification show that as the gender wage gap narrows, CSA perpetrated by mother’s partners declines significantly. However, there are several threats to the validity of these findings. For example, ${\bar{x}}_{jc}$ can still be correlated with $\mu_{jc}$ due to unobserved time-varying confounders that may have been omitted (Bell & Jones, 2015; Gunasekara et al., 2014). If the significance of the relationship between wage ratio and CSA by mothers’ partners were simply a result of an influential omitted variable, one might expect to see a similar influence exerted upon the wage ratio coefficients for sexual abuse perpetrated by biological fathers and/or family members, but that is not the case. Nonetheless, we re-estimate the model with lagged dependent variables to check if any time varying effects that are not included in the main specification are potentially biasing the results. It is important to note that inclusion of a lagged dependent variable leads to bias as it violates the assumptions of the random component of the model being independent of explanatory variables. Since $\mu_{jc}$ affects $y_{jct-\text{1}}$, inclusion of the lagged dependent variable as an explanatory factor creates correlation between the independent variables and the error term. As such the results presented in model 1 of Table 3 are strictly meant to be evaluated as a check for omitted variable bias. As expected, both the within and between coefficients of the lagged dependent variable are significant. Their inclusion shrinks the within coefficient of wage ratio from 2.178 to 1.88 but remains significant at 5%.

There is evidence that models with link functions other than identity do not reliably partition out the within and between variance when subject specific means are non-linearly related to the error terms (Bell et al., 2019; Brumback et al., 2010). When this happens, estimates can be inconsistent. Although biases are usually small (Goetgeluk & Vansteelandt, 2008), it is worth checking the possibility of non-linearity given the attenuation in statistical significance of wage ratio. We re-estimate the models by including polynomial terms of the county specific means of the independent variables. We also substitute the demeaned variables with the original variables; therefore, the county specific means provide the contextual effect in this specification (Allison, 2014). The main idea is that if the coefficients are not very different from the main specification, it is indicative that bias is not a significant cause of concern. Model 2 of Table 3 presents the results of the alternate specification. Despite the inclusion of the polynomial terms the effect of wage ratio on child sexual abuse perpetrated by mother’s romantic partners retains the negative and significant relationship. The coefficient of wage ratio remains significant at a 1% level and similar in size to the main specification. Model 3 shows that pooled results from 25 multiply imputed models yield results that are consistent with the main model.

Finally, to confirm the effect is driven by intra-household bargaining, we re-estimate the models with total wages instead of the female/male wage ratio as the main independent variable. Results in model 4 demonstrate that total wages have no effect on child sexual abuse. This is consistent with previous studies that have analyzed the impact of exogenous changes in macro-economic factors and found that it is the gender specific improvements that leads to a reduction in violence against women rather than a general improvement in overall conditions (Aizer, 2010; Anderberg et al., 2016). As a last check, we calculate rates of CSA perpetrated by mother’s romantic partners per 100,000 and estimate fixed effects model as well as a traditional mixed effects model with random intercepts for counties and state fixed effects. Results (in appendix) are robust to the alternate specifications.

### 4.2. Impact of Employment on Child Sexual Abuse

To distinguish the causal impact of difference forms of economic empowerment on bargaining power, we re-estimate the child sexual abuse model using the predicted employment rate as the main explanatory variable. Since it is the difference between the economic opportunities favoring men and women determines bargaining power (Anderberg et al., 2016), therefore we also operationalize the predicted employment rate as a ratio. Table 3 present the results of effect of employment on child sexual abuse.

Over time as the employment gap narrows, CSA exhibits an insignificant decline in model 1, 2, and 4. This insignificance supports that bargaining mechanism that suggests that wages and not employment increase bargaining power. Model 1 and 2 in Table 4 shows that on average counties with higher female employment rates have higher levels of CSA by biological fathers and mothers’ partners, but the effect is not significant. Previous studies that have found significant positive effects of gender specific employment on child maltreatment attribute it to increased exposure to abusive adults as mother increase the time contribution to paid work (Lindo et al., 2018).

## 5. Discussion

Exposure to childhood sexual abuse heightens the risk of developing various behavioral, mental, and physical problems (Dye, 2018; Guerra et al., 2018). Fortunately, there has been a significant decline in sexual victimization of children since the early 90s. While previous research has determined that this decline is not merely an artifact of measurement (Finkelhor et al., 2010), strategies credited for this prevention fail to provide robust evidence of effectiveness. Our study offers an alternate explanation using the intra-household bargaining framework and highlights an important protective mechanism through which this reduction may be taking place. Previous research shows that as improvement in economic prospects increase female bargaining power, not only do women face a lower risk of violence within relationships, but it is also easier for them to leave abusive partnerships. We argue that this effect extends to protect children from abusive individuals and provide evidence of a causal relationship between the gender wage gap and police reported CSA. We compare this effect across several different relationship categories which should not be impacted by changes in bargaining power. The results show no evidence of a decline in CSA perpetrated by strangers and family members in response to changes in gender specific wages. The findings are robust to sensitivity checks and provide empirical evidence that suggests that bargaining effect is indeed driving the reduction in CSA. This is consistent with previous literature that shows that child outcomes improve when mothers have more bargaining power and provide an additional rationale for gender wage parity. While it is outside the scope of the current study to analyze these specific mechanisms, it is difficult to conceive of a process whereby female autonomy and empowerment will lead to lower reporting mechanistically and result in an artificial relationship.

Our findings identify an important policy tool that can mitigate the burden of adverse childhood experiences. Mothers can play a significant role in primary prevention and be instrumental in inhibiting victimization of children. This is important because despite being one of the top preventable risk factors in the US burden of disease, CSA prevention is hampered by the fact that preemptive identification of prospective victims is challenging and resource intensive. Interventions aimed at education and disclosure alone are not sufficient and subsequent criminal justice actions to apprehend and prosecute perpetrators do not undo the lifetime consequences of trauma borne by the victims. Endowing mothers with the agency and capacity to be protective barriers can not only address the gaps in existing strategies but also have important spillover benefits operationalized through child wellbeing. It is important to note that closing the gender wage gap has no impact on CSA perpetrated by biological fathers. This may be due to limitations in the bargaining mechanism earlier and such a relationship may be better analyzed in the context of child custody laws.

We acknowledge that there are several limitations to this study. First, the data is anonymized, therefore, prevalence and incidence cannot be analyzed separately. Second, NIBRS is unlikely to capture the true incidence of child abuse and likely to suffer from underreporting problems. However, this is also true for alternate sources of child abuse data (Fallon et al., 2010). Despite these limitations, NIBRS data provides some distinct advantages through identification of the gender and relationship between the victim and offender and allowing for longitudinal analysis and is less likely to suffer from definitional variations that can impact other administrative data sources (Adhia, 2018).

Our results also show that different measures of improvements in women’s economic status are not equivalent in their protective effects and this distinction has important policy implications. As demonstrated, safety and well-being is achieved through good well-paying jobs rather than being merely employed. In addition to the direct and protective effect, closing the gender wage gap also has important spillover benefits. Larger effects transmitted via children’s well-being and their future productivity in the labor force can be substantial and should be considered explicitly in policy decisions. Although our study provides novel evidence on the relationship between CSA and macro-economic factors, it is merely highlighting a new path of inquiry. Future research should explore the relationship between macro-economic factors and CSA using multiple data sources and build on this initial evidence further. Scholars should also take into consideration the distinction between pertinent measures of bargaining power due to important policy implications.

## Supplementary Material

Supplement 1

## Figures and Tables

**Table 1:** Descriptive Statistics for Child Sexual Abuse Incidents

Variable	N	Mean	Std. dev.	Minimum	Maximum
CSA by Biological Fathers	2338	4.404	5.469	0	43
CSA by Mother’s Romantic Partners	2338	2.771	4.069	0	38
CSA by Family Members	2338	10.025	14.248	0	191
CSA by Strangers	2338	2.289	7.235	0	115
IPV Assaults	2338	1789.915	3623.4	0	40817
Gender Wage Ratio	2338	0.755	0.122	0.528	1.268
Gender Employment Ratio	2324	0.882	0.178	0.305	1.598
Secular Violence	2338	507.622	1342.12	1	16102
Yost Index	2310	10000.19	1079.439	8237	11855
Total Police Officer	2338	234.658	374.361	5	3454
Total SNAP Recipients	2338	13606.48	21168.21	604	266259
In-Migration	2338	6305.883	9078.927	309	99162
Total Child Population	2338	25254.8	36717.36	1298	286172
Percentage of Population - White	2338	0.752	0.193	0.102	0.991
Percentage of Population - Black	2338	0.222	0.190	0.004	0.883
Percentage of Population - Other	2338	0.025	0.026	0.003	0.224
Pct Men with Bachelor Degree or Higher	1360	0.189	0.102	0.0467	0.729
Pct Women with Bachelor Degree or Higher	1360	0.202	0.0952	0.077	0.738
Pct Female Headed Households with Minor Children	1360	0.022	0.019	0.001	0.126
Pct Households with Language Barrier	1360	0.697	0.021	0.012	0.137

Notes: Measures of intimate partner violence and secular are county year aggregates from 2006–2019 NIBRS data for South Carolina, Tennessee, and Virginia. Population counts and Yost index are from the Surveillance, Epidemiology and End Results Program. Number of total police officers are from the FBI’s LEOKA files and county level Migration data is from Internal Revenue Service. Wage and Employment data is from the Bureau of Economic Analysis and Bureau of Labor Statistics. Demographic data is from American Community Survey

**Table 2:** Impact of Wages on Child Sexual Abuse – By Relationship to Perpetrator

Model	(1)		(2)		(3)		(4)	
	Biological Fathers		Mothers’ Partner		Family Members		Strangers	
	Between	Within	Between	Within	Between	Within	Between	Within
Wage Ratio	−0.227	−0.496	−0.223	−2.192**	−0.485	−0.243	−1.242*	−0.610
	(0.321)	(0.619)	(0.329)	(0.719)	(0.276)	(0.441)	(0.488)	(0.970)
Yost Index	2.640**	−1.182	2.390*	−1.013	3.100***	−0.412	1.430	3.626*
	(0.908)	(1.281)	(0.929)	(1.437)	(0.775)	(0.866)	(1.384)	(1.766)
IPV	0.293*	0.328***	0.363**	0.195	0.220*	0.336***	0.023	0.104
	(0.123)	(0.093)	(0.126)	(0.112)	(0.104)	(0.066)	(0.186)	(0.145)
Secular Violence	0.454**	0.276***	0.286	0.336***	0.523***	0.243***	1.183***	0.736***
	(0.156)	(0.084)	(0.157)	(0.099)	(0.133)	(0.058)	(0.227)	(0.130)
SNAP Recipients	0.231	0.853***	0.435**	0.288	0.107	0.375**	−0.343	0.084
	(0.152)	(0.196)	(0.154)	(0.232)	(0.131)	(0.141)	(0.225)	(0.257)
Total Officers	−0.087	0.076	0.018	0.225	−0.023	0.106	0.196	−0.080
	(0.146)	(0.161)	(0.148)	(0.201)	(0.124)	(0.127)	(0.222)	(0.241)
In-migration	−0.227	0.010	−0.372**	0.144	−0.235*	0.148	−0.133	−0.158
	(0.127)	(0.127)	(0.131)	(0.147)	(0.108)	(0.088)	(0.190)	(0.181)
Percent Black	−0.020***	−0.018	−0.019***	−0.035	−0.010***	−0.007	−0.004	0.005
	(0.003)	(0.018)	(0.003)	(0.021)	(0.002)	(0.012)	(0.004)	(0.024)
Percent Other	−0.013	0.007	0.004	0.086	−0.033*	0.029	0.044	0.188**
	(0.019)	(0.042)	(0.019)	(0.056)	(0.016)	(0.032)	(0.028)	(0.059)
Constant	−27.129***		−25.526**		−30.072***		−15.994	
	(7.907)		(8.096)		(6.772)		(12.032)	
State *Year Effects	Yes		Yes		Yes		Yes	
Observations	2,310		2,310		2,310		2,310	
Number of Counties	165		165		165		165	
Log Likelihood	−4669.68		−41341		−6049.63		−2826.76	

Standard errors in parentheses

*** p < 0.001

** p < 0.01

* p < 0.05

Notes: Estimates are based on a random effect multi-level negative binomial model where outcome variable is aggregated county-year measure of sexual assaults perpetrated against children under 18 by fathers, mothers’ romantic partners, family members and strangers. Sexual assault includes forcible rape, forcible sodomy, sexual assault with an object, forcible fondling. The main variable of interest is the ratio of female and male wages.

County specific means of independent variables are included to control for fixed effects. Within effects represent the change in demeaned independent variables over time.

**Table 3:** Impact of Wage Ratio on Child Sexual Abuse Perpetrated by Mothers’ Partners – Sensitivity Analysis

Model	(1)		(2)		(3)		(4)	
	REWB Model		Contextual Model		Pooled REWB		REWB Model	
	Between	Within	Between	Original	Between	Within	Between	Within
Wage Ratio	−0.100	−1.882*	−10.249	−2.175**	−0239	−2.236**	-	-
	(0.304)	(0.786)	(22.295)	(0.720)	(0.300)	(0.710)	-	-
Yost Index	1.228	−0.185	63.641	−0.936	1.952*	−1.094	2.230*	−0.920
	(0.880)	(1.498)	(100.059)	(1.426)	(0.849)	(1.423)	(0.935)	(1.447)
IPV	0.298*	0.151	0.427	0.192	0.354**	0.161	0.367**	0.214
	(0.117)	(0.116)	(2.706)	(0.111)	(0.115)	(0.108)	(0.126)	(0.112)
Secular Violence	0.239	0.336**	0.037	0.339***	0.222	0.359***	0.286	0.333***
	(0.145)	(0.104)	(0.186)	(0.099)	(0.144)	(0097)	(0.158)	(0.099)
SNAP Recipients	0.270	0.243	20.793*	0.292	0.439**	0.295	0.425**	0.280
	(0.147)	(0.260)	(10.479)	(0.233)	(0.142)	(0.223)	(0.157)	(0.235)
Total Officers	−0.041	0.263	−5.311	0.226	0.046	0.207	0.013	0.216
	(0.137)	(0.213)	(3.434)	(0.199)	(0.136)	(0.195)	(0.150)	(0.203)
In-migration	−0.277*	0.153	16.616*	0.144	−0.275*	0.165	−0.371**	0.141
	(0.122)	(0.153)	(7.329)	(0.148)	(0.119)	(0.144)	(0.131)	(0.148)
Percent Black	−0.017***	−0.031	0.017	−0.036	−0.018***	−0.033	−0.020***	−0.034
	(0.003)	(0.024)	(0.022)	(0.022)	(0.003)	(0.021)	(0.003)	(0.021)
Percent Other	0.006	0.081	−0.111	0.089	−0.007	0.094	0.005	0.084
	(0.017)	(0.060)	(0.063)	(0.056)	(0.017)	(1.745)	(0.019)	(0.056)
Lagged CSA	0.056***	0.015***	-	-	-	-	-	-
	(0.012)	(0.003)	-	-	-	-	-	-
Total Wages							0.000	0.000
							(0.000)	(0.000)
Constant	−13.926		−420.086		22.2874		24.337**	
	(7.749)		(447.753)		(7.264)		(8.148)	
Polynomial Terms	-		Yes		-		-	
State *Year Effects	Yes		Yes		Yes		Yes	
Observations	2,145		2,310		2,310		2,310	
Number of Counties	165		165		165		165	
Log Likelihood	−3829.21		−4128.1		-		−4140.76	

Standard errors in parentheses

*** p < 0.01

** p < 0.05

* p < 0.1

Notes: Estimates are based on a random effect multi-level negative binomial model where outcome variable is aggregated county-year measure of police reported sexual assaults perpetrated against children under 18 by mothers’ romantic partners. Sexual assault includes forcible rape, forcible sodomy, sexual assault with an object, forcible fondling. The main variable of interest is the ratio of female and male wages. County specific means of independent variables are included to control for fixed effects. Within effects represent the change in demeaned independent variables over time.

**Table 4:** Impact of Employment Ratio on Child Sexual Abuse – By Relationship to Perpetrator

Model	(1)		(2)		(3)		(4)	
	Biological Fathers		Mothers’ Partner		Family Members		Strangers	
	Between	Within	Between	Within	Between	Within	Between	Within
Employment Ratio	0.045	−0.117	0.015	−0.072	−0.037	0.007	0.769	−0.341
	(0.252)	(0.246)	(0.260)	(0.286)	(0.216)	(0.172)	(0.401)	(0.420)
Yost Index	2.611**	−1.172	2.310*	−0.996	2.864***	−0.391	1.668	3.405
	(0.928)	(1.281)	(0.952)	(1.447)	(0.792)	(0.867)	(1.435)	(1.766)
IPV	0.288*	0.330**	0.361**	0.216	0.218*	0.337***	−0.037	0.119
	(0.125)	(0.093)	(0.127)	(0.112)	(0.105)	(0.065)	(0.190)	(0.144)
Secular Violence	0.452**	0.278***	0.282	0.331***	0.519***	0.244***	1.165***	0.745***
	(0.156)	(0.084)	(0.158)	(0.100)	(0.133)	(0.059)	(0.228)	(0.130)
SNAP Recipients	0.237	0.854***	0.442**	0.302	0.096	0.372**	−0.286	0.088
	(0.156)	(0.196)	(0.158)	(0.233)	(0.134)	(0.141)	(0.230)	(0.258)
Total Officers	−0.084	0.073	0.021	0.229	−0.013	0.106	0.273	−0.089
	(0.146)	(0.162)	(0.149)	(0.202)	(0.124)	(0.127)	(0.221)	(0.241)
In-migration	−0.226	0.009	−0.370**	0.147	−0.216*	0.148	−0.190	−0.157
	(0.129)	(0.127)	(0.133)	(0.148)	(0.110)	(0.088)	(0.196)	(0.180)
Percent Black	−0.021***	−0.017	−0.020***	−0.034	−0.011***	−0.007	−0.009*	0.002
	(0.003)	(0.018)	(0.003)	(0.021)	(0.002)	(0.012)	(0.004)	(0.024)
Percent Other	−0.012	0.007	0.005	0.083	−0.033*	0.030	0.053	0.188**
	(0.019)	(0.042)	(0.019)	(0.056)	(0.016)	(0.032)	(0.029)	(0.059)
Constant	−27.097***		−25.046**		−28.320***		−19.419	
	(8.170)		(8.375)		(6.999)		(12.593)	
State *Year Effects	Yes		Yes		Yes		Yes	
Observations	2,310		2,310		2,310		2,310	
Number of Counties	165		165		165		165	
Log Likelihood	−4654.21		−4034.1		−5930.63		−2802.76	

Standard errors in parentheses

*** p < 0.001

** p < 0.01

* p < 0.05

Notes: Estimates are based on a random effect multi-level negative binomial model where outcome variable is aggregated county-year measure of police reported sexual assaults perpetrated against children under 18 by fathers, mothers’ romantic partners, family members and strangers. Sexual assault includes forcible rape, forcible sodomy, sexual assault with an object, forcible fondling. The main variable of interest is the ratio of female and male wages. County specific means of independent variables are included to control for fixed effects. Within effects represent the change in demeaned independent variables over time.

## References

[R1] Adhia A. (2018). Predictors and consequences of intimate partner violence impacting children and youth [Doctoral dissertation, Harvard University].

[R2] Adhia A., Austin S. B., Fitzmaurice G. M., & Hemenway D. (2019). The role of intimate partner violence in homicides of children aged 2–14 years. American Journal of Preventive Medicine, 56(1), 38–46. 10.1016/j.amepre.2018.08.028 30416031

[R3] Ahmed M. (2006). Intra-household bargaining and investment in child health. In Union for African population studies fifth African population conference.2006.

[R4] Aizer A. (2010). The gender wage gap and domestic violence. American Economic Review, 100(4), 1847–1859. 10.1257/aer.100.4.1847 25110354 PMC4123456

[R5] Allison P. D. (2005). Fixed effects regression methods for longitudinal data using SAS. SAS Institute.

[R6] Allison P. D. (2009). Fixed effects regression models. SAGE Publications.

[R7] Allison P. D. (2014, September 2). Problems with the hybrid method. Statistical Horizons.Retrieved January 10, 2022, from https://statisticalhorizons.com/problems-with-the-hybrid-method/

[R8] Anderberg D., Rainer H., Wadsworth J., & Wilson T. (2016). Unemployment and domestic violence: Theory and evidence. The Economic Journal, 126(597), 1947–1979. 10.1111/ecoj.12246

[R9] Assink M., van der Put C. E., Meeuwsen M., de Jong N. M., Oort F. J., Stams G., & Hoeve M. (2019). Risk factors for child sexual abuse victimization: A meta-analytic review. Psychological Bulletin, 145(5), 459–489. 10.1037/bul0000188 30777768

[R10] Autor D. H., & Duggan M. G. (2003). The rise in the disability rolls and the decline in unemployment. The Quarterly Journal of Economics, 118(1), 157–206. 10.1162/00335530360535171

[R11] Bell A., Fairbrother M., & Jones K. (2019). Fixed and random effects models: Making an informed choice. Quality & Quantity, 53(2), 1051–1074. 10.1007/s11135-018-0802-x

[R12] Bell A., & Jones K. (2015). Explaining fixed effects: Random effects modeling of time-series cross-sectional and panel data. Political Science Research and Methods, 3(1), 133–153. 10.1017/psrm.2014.7

[R13] Berger L. M. (2005). Income, family characteristics, and physical violence toward children. Child Abuse & Neglect, 29(2), 107–133. 10.1016/j.chiabu.2004.02.006 15734178

[R14] Berger L. M., Paxson C., & Waldfogel J. (2009). Income and child development. Children and Youth Services Review, 31(9), 978–989. 10.1016/j.childyouth.2009.04.013 20368763 PMC2847736

[R15] Beyer K., Wallis A. B., & Hamberger L. K. (2015). Neighborhood environment and intimate partner violence: A systematic review. Trauma Violence Abuse, 16(1), 16–47. 10.1177/1524838013515758 24370630 PMC4476540

[R16] Bhattacharyya M., Bedi A. S., & Chhachhi A. (2011). Marital violence and women’s employment and property status: Evidence from north Indian villages. World Development, 39(9), 1676–1689. 10.1016/j.worlddev.2011.02.001

[R17] Bidarra Z. S., Lessard G., & Dumont A. (2016). Co-occurrence of intimate partner violence and child sexual abuse: Prevalence, risk factors and related issues. Child abuse & neglect, 55, 10–21. 27060785 10.1016/j.chiabu.2016.03.007

[R18] Bound J., & Holzer H. J. (2000). Demand shifts, population adjustments, and labor market outcomes during the 1980s. Journal of Labor Economics, 18(1), 20–54. 10.1086/209949

[R19] Brown D., & De Cao E. (2020). Child maltreatment, unemployment, and safety nets. SSRN Electronic Journal. 10.2139/ssrn.3543987

[R20] Brumback B. A., Dailey A. B., Brumback L. C., Livingston M. D., & He Z. (2010). Adjusting for confounding by cluster using generalized linear mixed models. Statistics & Probability Letters, 80(21–22), 1650–1654. 10.1016/j.spl.2010.07.006

[R21] Bullinger L. R., & Fong K. (2021). Evictions and neighborhood child maltreatment reports. Housing Policy Debate, 31(3–5), 490–515. 10.1080/10511482.2020.1822902

[R22] Bullinger L. R., Raissian K. M., & Schneider W. (2022). The power of the future: Intergenerational income mobility and child maltreatment in the United States. Child Abuse & Neglect, 130(Pt 4), 105175. 10.1016/j.chiabu.2021.105175 34266688

[R23] Bullinger L. R., & Ward B. C. (2021). What about the children? How opioid use affects child well-being. Contemporary Economic Policy, 39, 737–759. 10.1111/coep.12523

[R24] Cancian M., Yang M.-Y., & Slack K. S. (2013). The effect of additional child support income on the risk of child maltreatment. Social Service Review, 87(3), 417–437. 10.1086/671929

[R25] Craven S., Brown S., & Gilchrist E. (2006). Sexual grooming of children: Review of literature and theoretical considerations. Journal of Sexual Aggression, 12(3), 287–299. 10.1080/13552600601069414

[R26] Currie J., & Tekin E. (2012). Understanding the cycle: Childhood maltreatment and future crime. The Journal of Human Resources, 47(2), 509–549. 10.1353/jhr.2012.0017 24204082 PMC3817819

[R27] Dye H. (2018). The impact and long-term effects of childhood trauma. Journal of Human Behavior in the Social Environment, 28(3), 381–392. 10.1080/10911359.2018.1435328

[R28] Echeverría L., Menon M., Perali F., & Berges M. (2019). Intra-household inequality and child welfare in Argentina. Universidad Nacional de La Plata.

[R29] Edwards F. (2019). Family surveillance: Police and the reporting of child abuse and neglect. RSF: The Russell Sage Foundation Journal of the Social Sciences, 5(1), 50–70. 10.7758/rsf.2019.5.1.03

[R30] Fallon B., Trocme N., Fluke J., MacLaurin B., Tonmyr L., & Yuan Y., (2010). Methodological challenges in measuring child maltreatment. Child abuse & neglect, 34(1), 70–79 20053453 10.1016/j.chiabu.2009.08.008

[R31] Finkelhor D. (2009). The prevention of childhood sexual abuse. Future Child, 19(2), 169–194. 10.1353/foc.0.0035 19719027

[R32] Finkelhor D. (2020). Trends in adverse childhood experiences (ACEs) in the United States. Child Abuse & Neglect, 108, 104641. 10.1016/j.chiabu.2020.104641 32739600

[R33] Finkelhor D., & Ormrod R. (2001). Child abuse reported to the police. Juvenile Justice Bulletin. US Government Printing Office.

[R34] Finkelhor D., Saito K., & Jones L. (2018). Updated trends in child maltreatment, 2016. University of New Hampshire, Crimes against Children Research Center.

[R35] Finkelhor D., Turner H., Ormrod R., & Hamby S. L. (2010). Trends in childhood violence and abuse exposure: Evidence from 2 National Surveys. Archives of Pediatrics and Adolescent Medicine, 164(3), 238–242. 10.1001/archpediatrics.2009.283 20194256

[R36] Finkelhor D., Turner H. A., Shattuck A., & Hamby S. L. (2015). Prevalence of childhood exposure to violence, crime, and abuse: Results from the national survey of children’s exposure to violence. JAMA Pediatrics, 169(8), 746–754. 10.1001/jamapediatrics.2015.0676 26121291

[R37] Frioux S., Wood J. N., Fakeye O., Luan X., Localio R., & Rubin D. M. (2014). Longitudinal association of county-level economic indicators and child maltreatment incidents. Maternal and Child Health Journal, 18(9), 2202–2208. 10.1007/s10995-014-1469-0 24682605 PMC4180005

[R38] Fry D., & Elliott S. (2017). Understanding the linkages between violence against women and violence against children. The Lancet Global Health, 5(5), e472–e473. 10.1016/S2214-109X(17)30153-5 28395834

[R39] Fry D., Fang X., Elliott S., Casey T., Zheng X., Li J., Florian L., & McCluskey G. (2018). The relationships between violence in childhood and educational outcomes: A global systematic review and meta-analysis. Child Abuse & Neglect, 75, 6–28. 10.1016/j.chiabu.2017.06.021 28711191

[R40] Goetgeluk S., & Vansteelandt S. (2008). Conditional generalized estimating equations for the analysis of clustered and longitudinal data. Biometrics, 64(3), 772–780. 10.1111/j.1541-0420.2007.00944.x 18047524

[R41] Gorman M. F., & Ruggiero J. (2009). Evaluating US judicial district prosecutor performance using DEA: Are disadvantaged counties more inefficient? European Journal of Law and Economics, 27(3), 275–283. 10.1007/s10657-008-9093-3

[R42] Guerra C., Farkas C., & Moncada L. (2018). Depression, anxiety and PTSD in sexually abused adolescents: Association with self-efficacy, coping and family support. Child Abuse & Neglect, 76, 310–320. 10.1016/j.chiabu.2017.11.013 29179084

[R43] Gunasekara F. I., Richardson K., Carter K., & Blakely T. (2014). Fixed effects analysis of repeated measures data. International Journal of Epidemiology, 43(1), 264–269. 10.1093/ije/dyt221 24366487

[R44] Heckert J., Olney D. K., & Ruel M. T. (2019). Is women’s empowerment a pathway to improving child nutrition outcomes in a nutrition-sensitive agriculture program?: Evidence from a randomized controlled trial in Burkina Faso. Social Science & Medicine, 233, 93–102. 10.1016/j.socscimed.2019.05.016 31195195 PMC6642337

[R45] Hoynes H. W. (2000). Local labor markets and welfare spells: Do demand conditions matter? Review of Economics and Statistics, 82(3), 351–368. 10.1162/003465300558812

[R46] Jedwab M., Harrington D., & Dubowitz H. (2017). Predictors of substantiated re-reports in a sample of children with initial unsubstantiated reports. Child Abuse & Neglect, 69, 232–241. 10.1016/j.chiabu.2017.04.031 28486160

[R47] Johnson B. D. (2006). The multilevel context of criminal sentencing: Integrating judge‐and county‐level influences. Criminology, 44(2), 259–298. 10.1111/j.1745-9125.2006.00049.x

[R48] Kaplan J. (2021). National incident-based reporting system (NIBRS) data: A practitioner’s guide. [Computer software manual]. Retrieved January 2, 2021 from https://nibrsbook.com

[R49] Katz C., & Barnetz Z. (2016). Children’s narratives of alleged child sexual abuse offender behaviors and the manipulation process. Psychology of Violence, 6(2), 223–232. 10.1037/a0039023

[R50] Kohl P. L., Jonson-Reid M., & Drake B. (2009). Time to leave substantiation behind: Findings from a national probability study. Child Maltreatment, 14(1), 17–26. 10.1177/1077559508326030 18971346

[R51] Lindo J. M., Schaller J., & Hansen B. (2018). Caution! Men not at work: Gender-specific labor market conditions and child maltreatment. Journal of Public Economics, 163, 77–98. 10.1016/j.jpubeco.2018.04.007

[R52] Loughran T. A., Paternoster R., Chalfin A., & Wilson T. (2016). Can rational choice be considered a general theory of crime? Evidence from individual‐level panel data. Criminology, 54(1), 86–112. 10.1111/1745-9125.12097

[R53] Lundberg S. J., Pollak R. A., & Wales T. J. (1997). Do husbands and wives pool their resources? Evidence from the United Kingdom child benefit. The Journal of Human Resources, 32(3), 463–480. 10.2307/146179

[R54] Malapit H. J. L., Kadiyala S., Quisumbing A. R., Cunningham K., & Tyagi P. (2015). Women’s empowerment mitigates the negative effects of low production diversity on maternal and child nutrition in Nepal. The Journal of Development Studies, 51(8), 1097–1123. 10.1080/00220388.2015.1018904

[R55] Markowitz S., & Grossman M. (2000). The effects of beer taxes on physical child abuse. Journal of Health Economics, 19(2), 271–282. 10.1016/s0167-6296(99)00025-9 10947580

[R56] Mowbray O., Ryan J. P., Victor B. G., Bushman G., Yochum C., & Perron B. E. (2017). Longitudinal trends in substance use and mental health service needs in child welfare. Children and Youth Services Review, 73, 1–8. 10.1016/j.childyouth.2016.11.029

[R57] Nemeroff C. B. (2016). Paradise lost: The neurobiological and clinical consequences of child abuse and neglect. Neuron, 89(5), 892–909. 10.1016/j.neuron.2016.01.019 26938439

[R58] Paxson C., & Waldfogel J. (2002). Work, welfare, and child maltreatment. Journal of Labor Economics, 20(3), 435–474. 10.1086/339609

[R59] Pollak R. A. (2005). Bargaining power in marriage: Earnings, wage rates and household production. (NBER Working Paper No. 11239).

[R60] Raissian K. M. (2015). Does unemployment affect child abuse rates? Evidence from New York state. Child Abuse & Neglect, 48, 1–12. 10.1016/j.chiabu.2015.06.008 26238977

[R61] Raissian K. M., & Bullinger L. R. (2017). Money matters: Does the minimum wage affect child maltreatment rates? Children and Youth Services Review, 72, 60–70. 10.1016/j.childyouth.2016.09.033

[R62] Schneider W., Bullinger L. R., & Raissian K. M. (2021). How does the minimum wage affect child maltreatment and parenting behaviors? An analysis of the mechanisms. Review of Economics of the Household, 20, 1119–1154. 10.1007/s11150-021-09590-7

[R63] Slack K. S., Holl J. L., McDaniel M., Yoo J., & Bolger K. (2004). Understanding the risks of child neglect: An exploration of poverty and parenting characteristics. Child Maltreatment, 9(4), 395–408. 10.1177/1077559504269193 15538038

[R64] Staton-Tindall M., Sprang G., Clark J., Walker R., & Craig C. D. (2013). Caregiver substance use and child outcomes: A systematic review. Journal of Social Work Practice in the Addictions, 13(1), 6–31. 10.1080/1533256X.2013.752272

[R65] Thomas D. (1990). Intra-household resource allocation: An inferential approach. The Journal of Human Resources, 25(4), 635–664. 10.2307/145670

[R66] U.S. Department of Health & Human Services, Administration for Children and Families, Administration on Children, Youth and Families, & Children’s Bureau. (2019). Child maltreatment 2017. Children’s Bureau.

[R67] Wallace D., Chamberlain A., & Pfeiffer D. (2021). The relationship between foreclosures and intimate partner violence during the U.S. housing crisis. Journal of Interpersonal Violence, 36(13–14), 6247–6273. 10.1177/0886260518818431 30556475

[R68] Winters G. M., & Jeglic E. L. (2017). Stages of sexual grooming: Recognizing potentially predatory behaviors of child molesters. Deviant Behavior, 38(6), 724–733. 10.1080/01639625.2016.1197656

[R69] Wood J. N., Medina S. P., Feudtner C., Luan X., Localio R., Fieldston E. S., & Rubin D. M. (2012). Local macroeconomic trends and hospital admissions for child abuse, 2000–2009. Pediatrics, 130(2), e358–e364. 10.1542/peds.2011-3755 22802600

[R70] Yost K., Perkins C., Cohen R., Morris C., & Wright W. (2001). Socioeconomic status and breast cancer incidence in California for different race/ethnic groups. Cancer Causes Control, 12(8), 703–711. 10.1023/a:1011240019516 11562110

[R71] Younger J. W., Chu L. F., D’Arcy N. T., Trott K. E., Jastrzab L. E., & Mackey S. C. (2011). Prescription opioid analgesics rapidly change the human brain. Pain, 152(8), 1803–1810. 10.1016/j.pain.2011.03.028 21531077 PMC3138838

