# Supplementary material for: Gender Wage Gap and Male Perpetrated Child Sexual Abuse
Source: Res Sq. 2023 Apr 28:rs.3.rs-2857277. Preprint. [Version 1] doi: 10.21203/rs.3.rs-2857277/v1 (PMC10168437; doi:10.21203/rs.3.rs-2857277/v1)
Supplement: Supplement 1 [file NIHPPRS2857277V1-supplement-1.pdf]

Supplementary Files

This is a list of supplementary files associated with this preprint. Click to download.

- Appendix.docx
